# Lidocaine Alleviates Sepsis-Induced Acute Lung Injury in Mice by Suppressing Tissue Factor and Matrix Metalloproteinase-2/9

**DOI:** 10.1155/2021/3827501

**Published:** 2021-11-12

**Authors:** Binbin Zheng, Hongbo Yang, Jianan Zhang, Xueli Wang, Hao Sun, Fan Hu, Qian Li, Liping Jiang, Yue Su, Qilin Peng, Yulin Tang, Wen-Tao Liu, Xueming He, Yixin Fan, Xia Zhu

**Affiliations:** ^1^Center for Clinical Research and Translational Medicine, The Affiliated Lianyungang Oriental Hospital of Xuzhou Medical University, Lianyungang 222042, China; ^2^Center for Clinical Research and Translational Medicine, The Affiliated Lianyungang Oriental Hospital of Bengbu Medical College, Lianyungang 222042, China; ^3^Center for Clinical Research and Translational Medicine, The Affiliated Lianyungang Oriental Hospital of Kangda College of Nanjing Medical University, Lianyungang 222042, China; ^4^Jiangsu Key Laboratory of Neurodegeneration, Department of Pharmacology, Nanjing Medical University, Nanjing 211166, China; ^5^Department of Emergency, Jiangsu Province Hospital, The First Affiliated Hospital of Nanjing Medical University, Nanjing 210029, China; ^6^Department of Anesthesiology, The Affiliated Jiangning Hospital of Nanjing Medical University, Nanjing, Jiangsu, China; ^7^Department of Pharmacy, Sir Run Run Hospital, Nanjing Medical University, No. 109 Longmian Avenue, Nanjing 211100, China

## Abstract

Acute lung injury (ALI) is one of the fatal symptoms of sepsis. However, there were no effective clinical treatments. TF accumulation-induced fibrin deposit formations and coagulation abnormalities in pulmonary vessels contribute to the lethality of ALI. Suppressor of cytokine signaling 3 (SOCS3) acts as an endogenous negative regulator of the TLR4/TF pathway. We hypothesized that inducing SOCS3 expression using lidocaine to suppress the TLR4/TF pathway may alleviate ALI. Hematoxylin and eosin (H&E), B-mode ultrasound, and flow cytometry were used to measure the pathological damage of mice. Gelatin zymography was used to measure matrix metalloproteinase-2/9 (MMP-2/9) activities. Western blot was used to assay the expression of protein levels. Here, we show that lidocaine could increase the survival rate of ALI mice and ameliorate the lung injury of ALI mice including reducing the edema, neutrophil infiltration, and pulmonary thrombosis formation and increasing blood flow velocity. Moreover, *in vitro* and *in vivo*, lidocaine could increase the expression of p-AMPK and SOCS3 and subsequently decrease the expression of p-ASK1, p-p38, TF, and the activity of MMP-2/9. Taken together, our study demonstrated that lidocaine could inhibit the TLR4/ASK1/TF pathway to alleviate ALI via activating AMPK-SOCS3 axis.

## 1. Introduction

Acute lung injury (ALI) caused by sepsis is one of the main causes of death in ICU patients. Unfortunately, therapies to prevent or treat ALI remain elusive. It is urgent to further investigate the pathogenesis of sepsis-induced ALI and find effective strategy.

The pathological characteristics of ALI mainly include coagulation abnormalities, thrombus, hypoxemia, diffuse infiltration of the lungs, and decreased functional lung volume [1]. Tissue factor (TF) is a primary trigger for coagulation and thrombus formation, playing a pivotal role in blood clotting activation [2]. Recent study showed that the expression of tissue factor (TF) was significantly increased in LPS-induced ALI model in mice, leading to a hypercoagulable state in the pulmonary vasculature [3]. TF expressed in the microvasculature acts as a critical initiator of blood coagulation in ALI, while using the neutralizing antibody to inhibit TF before lethal sepsis prevents ALI in baboons [4, 5]. In addition, study found that antithrombin (hirudin) could also have a protective effect on endotoxemia mice by reducing fibrin deposition [6]. These studies indicated that moderate anticoagulation has certain therapeutic effect on sepsis-induced ALI.

Apoptosis signal-regulating kinase (ASK1) is a member of the MAP3K family, which is activated by a number of cellular stressors, such as oxidative stress and inflammatory cytokines [7, 8]. Study found that ASK1 activation could significantly induce the expression of TF [9], participating in regulation of hemostasis and thrombosis [10]. As the upstream signaling molecule of ASK1, Toll-like receptor 4 (TLR4) plays an important role in the activation of innate immunity, inducing the production of IL-6, TNF-*α*, and even TF through the p38/NF-*κ*B signaling [11–13]. Thus, inhibition of the TLR4/ASK1 signaling pathway may be beneficial to ALI. Suppressor of cytokine signaling 3 (SOCS3) is an inhibitor of cytokine signaling pathways, which have been found that can negatively regulate the TLR4-mediated signaling pathway [14], and our previous study also found that inducing the overexpression of SOCS3 can inhibit the activation of the TLR4-p38 pathway [15]. Taken together, upregulating SOCS3 expression to inhibit TLR4-ASK1 axis and decrease TF expression may be an effective strategy to alleviate ALI.

Lidocaine is a common amide-type local anesthetic. Recent study indicated that local anesthetics using lidocaine could inhibit LPS-induced tissue factor messenger RNA (TF mRNA) level in endothelial and monocytes, but the underlying mechanism is unclear [16]. Our previous study has shown that lidocaine could upregulate the expression of SOCS3 and inhibit the activation of TLR4 signal, as well as suppress neuroinflammation [17]. Given the above discussion, we hypothesized lidocaine could induce SOCS3 production in the lungs, decreasing TF expression and alleviating LPS-induced ALI.

## 2. Materials and Methods

### 2.1. Chemicals and Reagents

Lidocaine was purchased from Hubei Tianyao Pharmaceutical Co. Ltd. LPS (catalog no. L2630) and compound C (catalog no. P5499) were purchased from Sigma-Aldrich (St. Louis, MO, USA). Antibody for SOCS3 (catalog no. ab16030), HIF-1*α* (catalog no. ab1), and TF (catalog no. ab189483) was purchased from Abcam (Cambridge, MA, USA). Antibody for phosphorylated AMP-activated protein kinase (p-AMPK) (Thr172; catalog no. 2531), phosphorylated ASK1 (catalog no. 3762S), and phosphorylated p38 mitogen-activated protein kinase (p38; Tyr180/Tyr182; catalog no. 9211) was purchased from Cell Signaling Technology (Beverly, MA, USA). Antibody for *β*-actin (catalog no. A1978) was purchased from Sigma-Aldrich. Fetal bovine serum (FBS) was purchased from Gibco, and other cell culture media and supplements were purchased from KenGen (KenGen Biotech, China). All other reagents were from Sigma-Aldrich (St. Louis, MO, USA).

### 2.2. Animals and Treatment

Adult male CD-1 mice (18-22 g) were purchased from the Experimental Animal Center at Nanjing Medical University, Nanjing, China. Animals were housed five to six per cage under pathogen-free conditions with soft bedding under controlled temperature (22 ± 2°C) and a 12 h light/dark cycle (lights on at 8 : 00 a.m.). Animals were allowed to acclimate to these conditions for at least 3 days before inclusion in experiments. For each group of experiments, the animals were matched by age and body weight. All procedures were conducted in accordance with the guidelines and regulations of the National Institutes of Health (NIH) and were approved by the Ethics Committee of Nanjing Medical University (No. IACUC-1902012).

The model of sepsis-induced ALI was induced by a single intraperitoneal (i.p.) injection of 12.5 mg/kg of LPS in mice as previously described [18, 19]. Mice were randomly divided into 6 groups (*n* = 12) according to treatment, including control group (Con), LPS group, lidocaine (2 mg/kg)+LPS group, lidocaine (4 mg/kg)+LPS group, lidocaine (8 mg/kg)+LPS group, and lidocaine (8 mg/kg) group. For the survival rate and body weight experiments, different concentrations of lidocaine (2, 4, and 8 mg/kg) were intravenous (i.v.) injection into mice through the tail vein every 12 h for 3 consecutive days. For the other experiments, lidocaine (2, 4, and 8 mg/kg) was i.v. injection into mice through the tail vein 30 min before LPS injection.

### 2.3. Bronchial Alveolar Lavage Fluid (BALF) Collection and Measurement

The methods to collect BALF were described previously [20]. After LPS injection for 12 h, the mice were anesthetized by 1% sodium amobarbital, and then, the thorax was opened. 500 *μ*L of PBS was injected, retrieved the liquid from the trachea, and repeated three times. The samples were centrifuged at 5000 rpm for 10 min, and the supernatants were collected. The total protein level in BALF was measured by the BCA kit (Thermo Scientific, MA, USA) according to the manufacturer's protocol.

### 2.4. Hematoxylin and Eosin (H&E)

For the histological analysis, the mice were anesthetized by 1% sodium amobarbital and sacrificed and then quickly collected the lung tissues from mice. The samples were fixed in 10% formalin for 24 h and then dehydrated in a series of graded ethanol and finally embedded in paraffin. Then, microtome sections (4 *μ*m) were cut and stained with hematoxylin and eosin (H&E). Images were taken under a light microscope (Leica, Germany) by two investigators blinded to a group assignment. Six random fields of each lung section were examined.

### 2.5. Lung Wet-to-Dry Ratio Assay

Lung wet-to-dry ratio was used as an index of lung edema. After LPS injection for 12 h, mice were sacrificed, and the lung tissues (both lobes) were immediately weighed after collection and recorded as wet weight. The lung tissues were then placed into an oven at 60°C for 72 h and weighed as dry weight. Lung wet-to-dry ratio was calculated by dividing the wet by the dry weights.

### 2.6. Ultrasonic Testing

The mice were anesthetized by 1% sodium amobarbital, and ultrasonic testing was performed by the US GE E9 ultrasonic machine and 11L ultrasonic probe. The test method was referred to Lichtenstein DA double blue dot + PLAPS point detection which checks the distribution of the B line and assesses the distribution area of the B line in the scanning area for semiquantitative analysis. In order to correct the measurement bias, a double-blind design was used during the ultrasonic detection. Neither the investigators nor the sonographers were aware of the type of the mouse group. Each mouse underwent ultrasonic detection for three times, and the detection was repeated by two sonographers with more than 3 years' experience. The average values of the two measurements were statistically analyzed.

### 2.7. Cell Cultures

Human mononuclear macrophages (THP-1, No. ZQ 0086) were purchased from the Shanghai Zhongqiao Biological Research Institute, China, and maintained in humidified 5% CO_2_ at 37°C in Dulbecco's modified Eagle's Medium (DMEM; KenGen Biotech, China) supplemented with 10% (*v*/*v*) FBS, penicillin (100 U/mL), and streptomycin (100 U/mL). For further experiments, 10^5^ cells were plated in a 6-well plate overnight and then treated with LPS (100 ng/mL) in the following morning with or without lidocaine for 12 h. Cell extracts and precipitated supernatants were collected and analyzed by immunoblot assay.

### 2.8. Gelatin Zymography

Mice were anesthetized with 1% sodium amobarbital and sacrificed, and then, the lung tissues were rapidly dissected and homogenized in 1% NP40 lysis. 250-300 *μ*g of protein per lane was loaded into the wells of precast gels (8% polyacrylamide gels containing 0.1% gelatin). After electrophoresis, each gel was incubated with 50 mL of developing buffer for 48 h (37°C) in shaking bath. Then, the gels were stained with Coomassie brilliant blue (1%, with 10% acetic acid, 10% isopropyl alcohol, diluted with ddH_2_O).

### 2.9. Western Blotting

The lung tissues and cells were collected and washed with ice-cold PBS before being lysed in radio immunoprecipitation assay (RIPA) lysis buffer. The protein concentrations were determined by BCA Protein Assay (Thermo Fisher, Waltham, MA), and 40-80 *μ*g of proteins were loaded and separated by SDS-PAGE and electrophoretically transferred onto polyvinylidene fluoride membranes (Millipore Corp., Bedford, MA). The membranes were blocked with 5% milk in TBST (Tris-HCl, NaCl, and Tween 20) for 2 h at room temperature and then probed with primary antibodies at 4°C for overnight. Finally, the horseradish peroxidase- (HRP-) coupled secondary antibodies were utilized for detecting corresponding primary antibody. The primary antibodies used included p-AMPK (Thr172) (1 : 1000), p-ASK1 (1 : 1000), SOCS3 (1 : 1000), p-p38 (1 : 1000), HIF-1*α* (1 : 1000), and TF (1 : 1000). For loading control, the blots were probed with antibody for *β*-actin (1 : 8000). The filters were then developed by enhanced chemiluminescence reagents (PerkinElmer, Waltham, MA) with secondary antibodies (Chemicon, Billerica, MA). Data were acquired with the Molecular Imager (Gel Doc^™^ XR, 170-8170) and analyzed with Quantity One-4.6.5 (Bio-Rad Laboratories, Berkeley, CA).

### 2.10. Flow Cytometry

Mice were anaesthetized deeply with 1% sodium amobarbital and then collected blood samples, and the red blood cell lysis buffer (Sigma-Aldrich, MO, USA) was added into the blood for 20 min, after centrifugation (1000 r/min, 5 min) and collected the cells, then added the 1% BSA into the cells, and centrifugation (1000 r/min) again for 5 min, and then the cells were collected and stained with monoclonal anti-mouse antibodies: CD45-PE, CD11b-APE, and Ly6G. Cells were stained for 40 min on ice before being washed and analyzed on a FACSVerse flow cytometer (BD, NJ, USA) using FlowJo V10.0.7 (Tree Star, Ashland, USA). CD45^+^ CD11b^+^ Ly6G^+^-positive cells were considered neutrophils.

### 2.11. Statistical Analyses

GraphPad Prism 6 software (GraphPad Software, San Diego, CA) was used to conduct all the statistical analyses. Kaplan-Meier survival analysis was completed using the log rank (Mantel-Cox) test. Data were statistically evaluated by one-way or two-way analysis of variance (ANOVA) followed by the Bonferroni post hoc tests. Results were represented as the mean ± SEM of three independent experiments. Results described as significant were based on a criterion of *P* < 0.05.

## 3. Results

### 3.1. Lidocaine Significantly Alleviates LPS-Induced ALI

**Figure 1:**
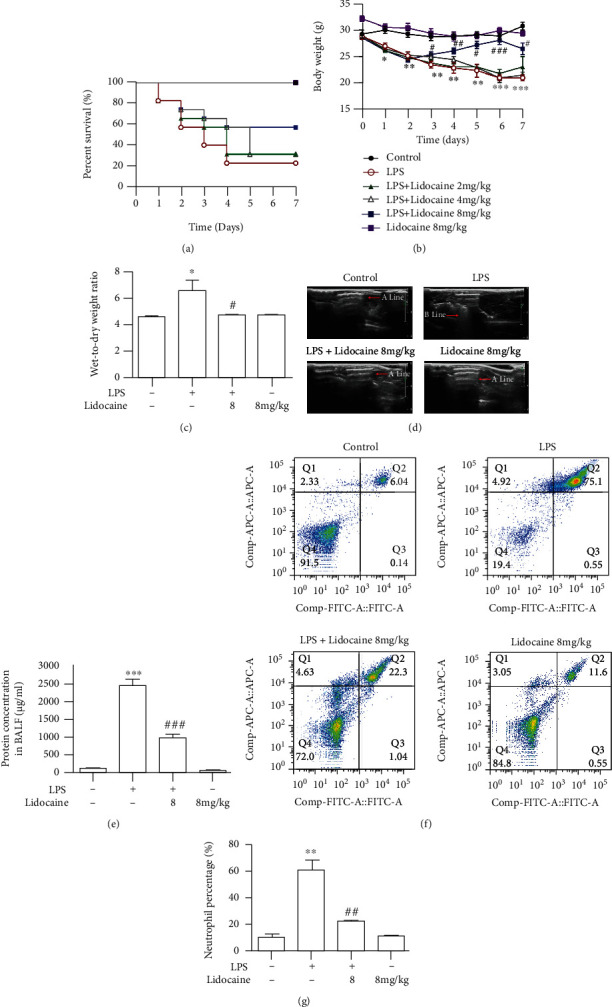
Lidocaine significantly alleviated sepsis-induced acute lung injury. Survival rate (a) and body weight (b) of mice were measured every day for 7 consecutive days (*n* = 12 of each group). (c) Wet/dry weight of mouse lung tissues was measured 12 h after intraperitoneal (i.p.) injection of 12.5 mg/kg LPS to mice. (d) Representative images of B-mode ultrasound. Normal mice show an “A” line, while “B” line indicates pulmonary edema in mice. (e) Total protein concentration in bronchoalveolar lavage fluid (BALF). (f) Neutrophil proportion in the blood assessed by flow cytometric analysis. (g) Quantitative data of the neutrophil proportion analyzed by flow cytometry. (*n* = 4 of each group). Significant difference was revealed following one-way or two-way ANOVA (^∗^*P* < 0.05, ^∗∗^*P* < 0.01, and ^∗∗∗^*P* < 0.001 vs. control; ^#^*P* < 0.05, ^##^*P* < 0.01, and ^###^*P* < 0.001 vs. LPS-treated group; Bonferroni post hoc tests).

As shown in Figure 1(a), mice administered with LPS (12.5 mg/kg, i.p.) showed decreased survival (20%) on the 7^th^ day as compared to the control group (100%), whereas preadministration of lidocaine (8 mg/kg, i.v.) could significantly increase LPS-treated mouse survival rate. Besides, compared with the LPS group, preadministration of lidocaine (8 mg/kg, i.v.) could also increase the weight of mice from 24.4 ± 0.52 g to 26.43 ± 1.11 g (Figure 1(b)). Moreover, it was found that LPS stimulation for 12 h significantly increased the ratio of wet-to-dry weight of mouse lung tissues from 4.52 ± 0.06 to 6.52 ± 0.76 (Figure 1(c)), and the B-mode ultrasound imaging also indicated that LPS significantly induce pulmonary edema in mice (Figure 1(d)), whereas these effects could be reversed by lidocaine (Figures 1(c) and 1(d)). Further study has found that in i.v. injection of 8 mg/kg of lidocaine into mice for 12 h, the plasma drug concentration reached the lung tissue of 370.3 ± 94.3 ng/mL (Figure S1 and Table S1). In addition, through total protein quantification and flow cytometry detection, it was found that the total protein content in BALF and the number of neutrophils in the blood of LPS-treated mice were significantly increased, and these effects could be reversed by lidocaine (Figures 1(e) and 1(g)), indicating that lidocaine could alleviate LPS-induced severe alveolar leakage. These data suggested that lidocaine could significantly alleviate sepsis-induced ALI.

### 3.2. Lidocaine Ameliorates LPS-Induced Pulmonary Coagulopathy

**Figure 2:**
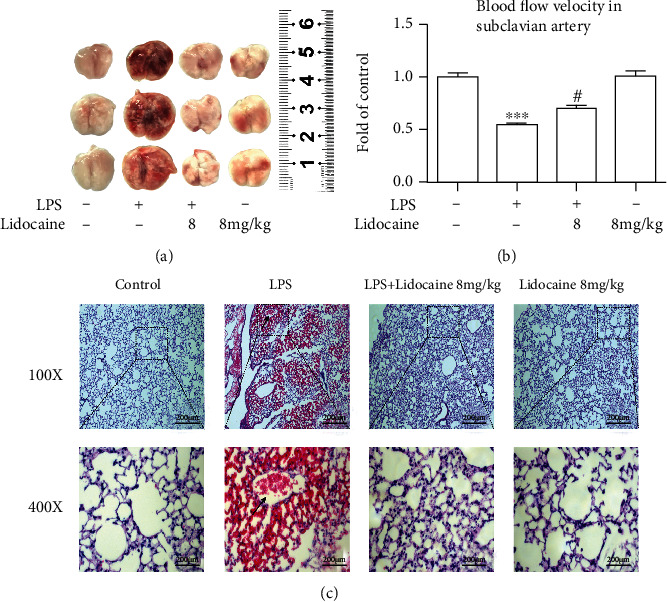
Lidocaine markedly ameliorated LPS-induced pulmonary coagulopathy. (a) Representative photographs of lung tissues 12 h after LPS injection. (b) Quantitative data of blood flow velocity in the subclavian artery. (c) Hematoxylin- and eosin-stained (HE) sections of the lung tissues of mice. Black arrow indicated thrombi. Magnifications: 100x and 400x (*n* = 3 of each group). Significant difference was revealed following one-way ANOVA (^∗∗∗^*P* < 0.001 vs. control; ^#^*P* < 0.05 vs. LPS-treated group; Bonferroni post hoc tests).

Many clinical studies showed that virtually most of septic patients had coagulation abnormalities. Therefore, in this study, we intend to investigate the effect of lidocaine on pulmonary coagulation function induced by LPS in mice. As shown in Figure 2(a), LPS obviously induced the production of thrombotic plaques in the lung tissue of mice, while preadministration of lidocaine (8 mg/kg, i.v.) could significantly reduce the formation of thrombus. In addition, compared with the LPS group, B-mode ultrasound detection also showed that lidocaine (8 mg/kg, i.v.) could increase the blood flow velocity in the subclavian artery in mice (Figure 2(b)). The hematoxylin and eosin (H & E) staining results demonstrated that LPS significantly induced lung tissue structural imperfection and alveolar wall thickening. Furthermore, many thrombi obstructing the pulmonary vasculature were visible in the histological sections, whereas lidocaine significantly ameliorated histopathological damage and reduced the production of pulmonary thrombosis (Figure 2(c)). Altogether, these results suggested that lidocaine could ameliorate LPS-induced pulmonary coagulation disorders.

### 3.3. Lidocaine Could Inhibit TF Expression and MMP-2/9 Activity Induced by LPS in Plasma

**Figure 3:**
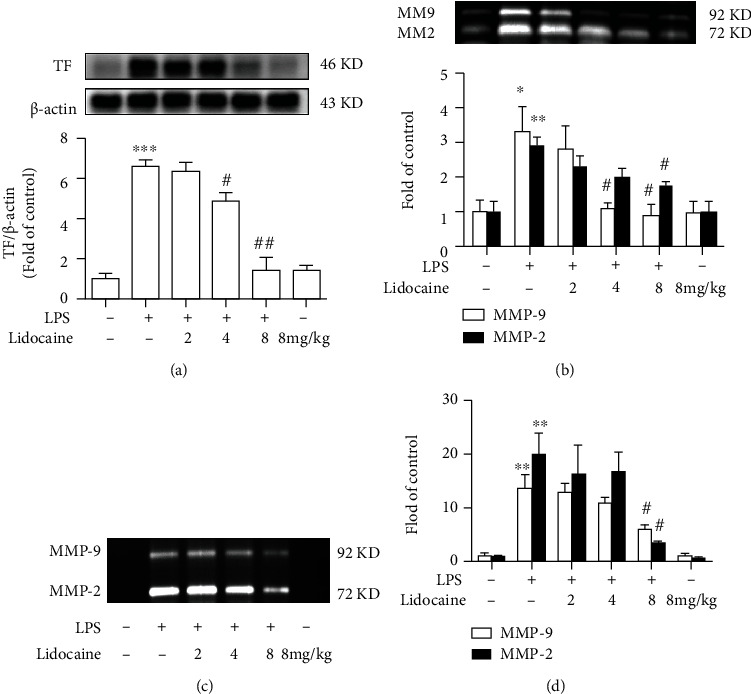
Lidocaine significantly suppressed LPS-induced increase of tissue factor (TF) and matrix metalloproteinase (MMP)-2/9 in plasma and BALF from mice. (a) Effect of lidocaine on the expression of TF in plasma was measured by Western blot analysis, and the densitometry values were normalized to *β*-actin. (b) Effect of lidocaine on the activity of MMP-2/9 in plasma was measured by gelatin zymography, and the densitometry values were normalized. (c, d) Representative image and densitometry showed the activity of MMP-2/9 in BALF. Mice were treated with different dosages of lidocaine (2, 4, and 8 mg/kg, i.v.) 30 min before LPS injection, and the plasma and BALF samples (*n* = 4 of each group) were collected 12 h after LPS given. Significant difference was revealed following one-way ANOVA (^∗^*P* < 0.05, ^∗∗^*P* < 0.01, and ^∗∗∗^*P* < 0.001 vs. control; ^#^*P* < 0.05 and ^##^*P* < 0.01 vs. LPS-treated group; Bonferroni post hoc tests).

Previous studies have found that the circulating blood in patients with sepsis presents a hypercoagulable state [21], and Shaver et al. have also found that the severity of LPS-induced ALI is positively correlated with the level of TF in the circulating blood in mice [22]. In this study, we detected TF expression and MMP-2/9 activity in the plasma of LPS-treated mice. As shown in Figures 3(a) and 3(b), compared with the control group, LPS (12.5 mg/kg, i.p.) significantly increased the expression level of TF, up to 6.6-fold, and the activities of MMP-2/9 in plasma were increased up to 2.92-fold and 3.33-fold, respectively, whereas preadministration of lidocaine (4 and 8 mg/kg, i.p.) could reverse the LPS-induced upregulation of TF and MMP-2/9 in mice (Figures 3(a) and 3(b)). In addition, lidocaine (8 mg/kg) could also significantly reduce the activity of MMP-2/9 in BALF of LPS-treated mice from 19.97-fold to 3.37-fold and from 13.76-fold to 5.87-fold, respectively (Figures 3(c) and 3(d)).

### 3.4. Lidocaine Could Significantly Inhibit LPS-Induced TF Expression and MMP-2/9 Activity in the Lungs

**Figure 4:**
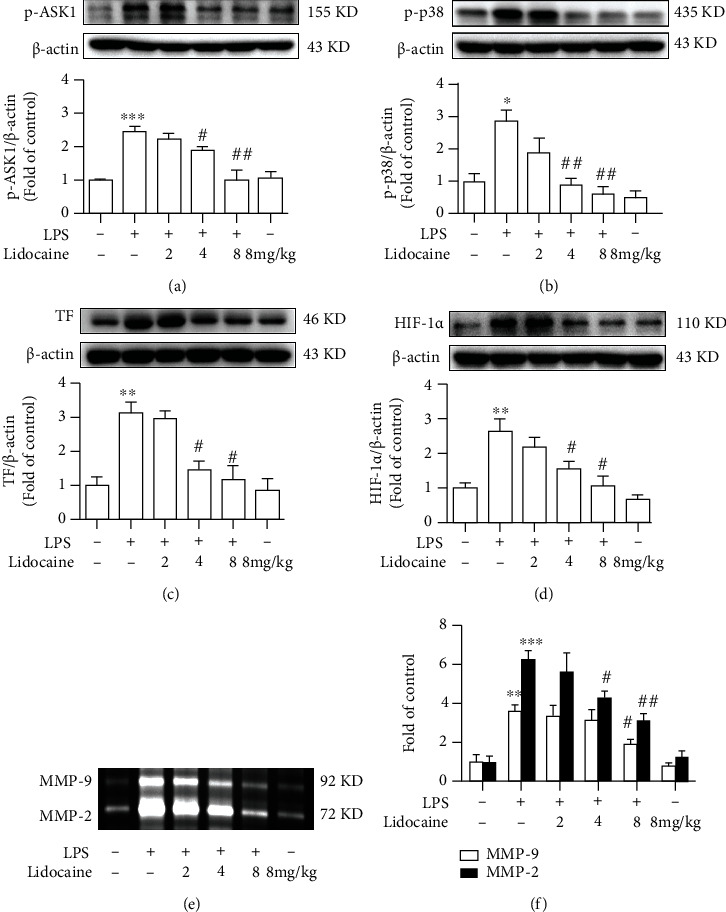
Lidocaine significantly inhibited LPS-induced expression of tissue factor (TF) and matrix metalloproteinase (MMP)-2/9 in the lung tissues from mice. Representative Western blot images showed the levels of p-ASK1 (a), p-p38 (b), TF (c), and HIF-1*α* (d) in lung tissues. (e, f) Activity of MMP-2/9 in lung tissues was measured by gelatin zymography, and the densitometry values were normalized. Mice were treated with different dosages of lidocaine (2, 4, and 8 mg/kg, i.v.) 30 min before LPS injection, and the tissue samples (*n* = 4 of each group) were collected 12 h after LPS given. Significant difference was revealed following one-way or two-way ANOVA (^∗^*P* < 0.05, ^∗∗^*P* < 0.01, and ^∗∗∗^*P* < 0.001 vs. control; ^#^*P* < 0.05 and ^##^*P* < 0.01 vs. LPS-treated group; Bonferroni post hoc tests).

We further investigated the signaling pathway of the LPS-induced high level of TF and MMP-2/9 in mice. It has been reported that ASK1 is crucial for the formation of thrombus [10], and ASK1 activation may also increase the transcription level of TF mRNA through the MAPK signaling [3]. Compared with the LPS group, LPS could significantly induce the upregulation of p-ASK1 and p-p38 in the lungs of mice up to 2.45-fold and 2.87-fold, respectively, while lidocaine (4 and 8 mg/kg, i.p.) significantly inhibited the activation of ASK1 and p38 (Figures 4(a) and 4(b)). In addition, LPS could also induce the expression of TF, HIF-1*α*, and MMP-2/9 in the lungs, whereas preadministration with 4 or 8 mg/kg of lidocaine could significantly inhibit these effects (Figures 4(c) and 4(f)). These data suggested that lidocaine could decrease the expression of TF, HIF-1*α*, and MMP-2/9 by inhibiting the ASK1-p38 signaling pathway.

### 3.5. Lidocaine Significantly Increased the Expression of p-AMPK and SOCS3 In Vitro and In Vivo

**Figure 5:**
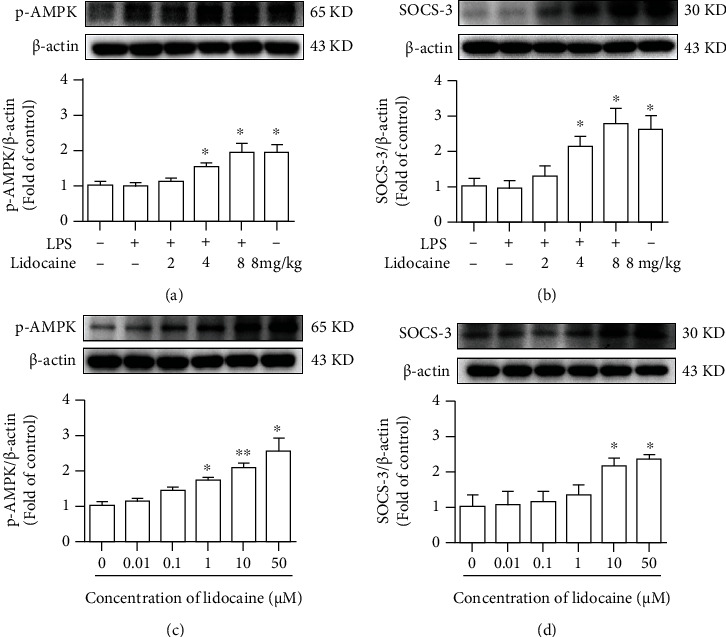
Lidocaine significantly induced expression of p-AMPK and SOCS3 both *in vivo* and *in vitro*. (a, b) Representative Western blot images showed the levels of p-AMPK and SOCS3 in lung tissues from mice given different dosages of lidocaine (2, 4, and 8 mg/kg, i.v.) 30 min before LPS injection (*n* = 4 of each group). (c, d) Representative Western blot bands have shown the level of p-AMPK and SOCS3 in THP1 cells cultured with different concentrations of lidocaine for 6 h. Significant difference was revealed following one-way ANOVA (^∗^*P* < 0.05 and ^∗∗^*P* < 0.01 vs. control; Bonferroni post hoc tests).

Our previous studies showed that lidocaine could induce the expression of SOCS3 and inhibit neuroinflammation in an AMPK-dependent manner [17]. Therefore, we also investigated whether lidocaine could activate AMPK and induce the production of SOCS3 in lung tissues of mice *in vivo*. Different concentrations of lidocaine (2, 4, and 8 mg/kg) were administrated to mice; data showed that lidocaine (4 and 8 mg/kg) significantly activated AMPK and induced SOCS3 expression *in vivo* (Figures 5(a) and 5(b)). Moreover, we also performed corresponding experiments on THP1 cells *in vitro*. THP1 cells were treated with different concentrations of lidocaine (from 0.01 to 50 *μ*M) for 12 h. As shown in Figures 5(c) and 5(d), lidocaine significantly increased the expression of p-AMPK and SOCS3 in a concentration-dependent manner *in vitro*.

### 3.6. Lidocaine Protects against LPS-Induced Inflammation via the AMPK/SOCS3 Signaling Pathway

**Figure 6:**
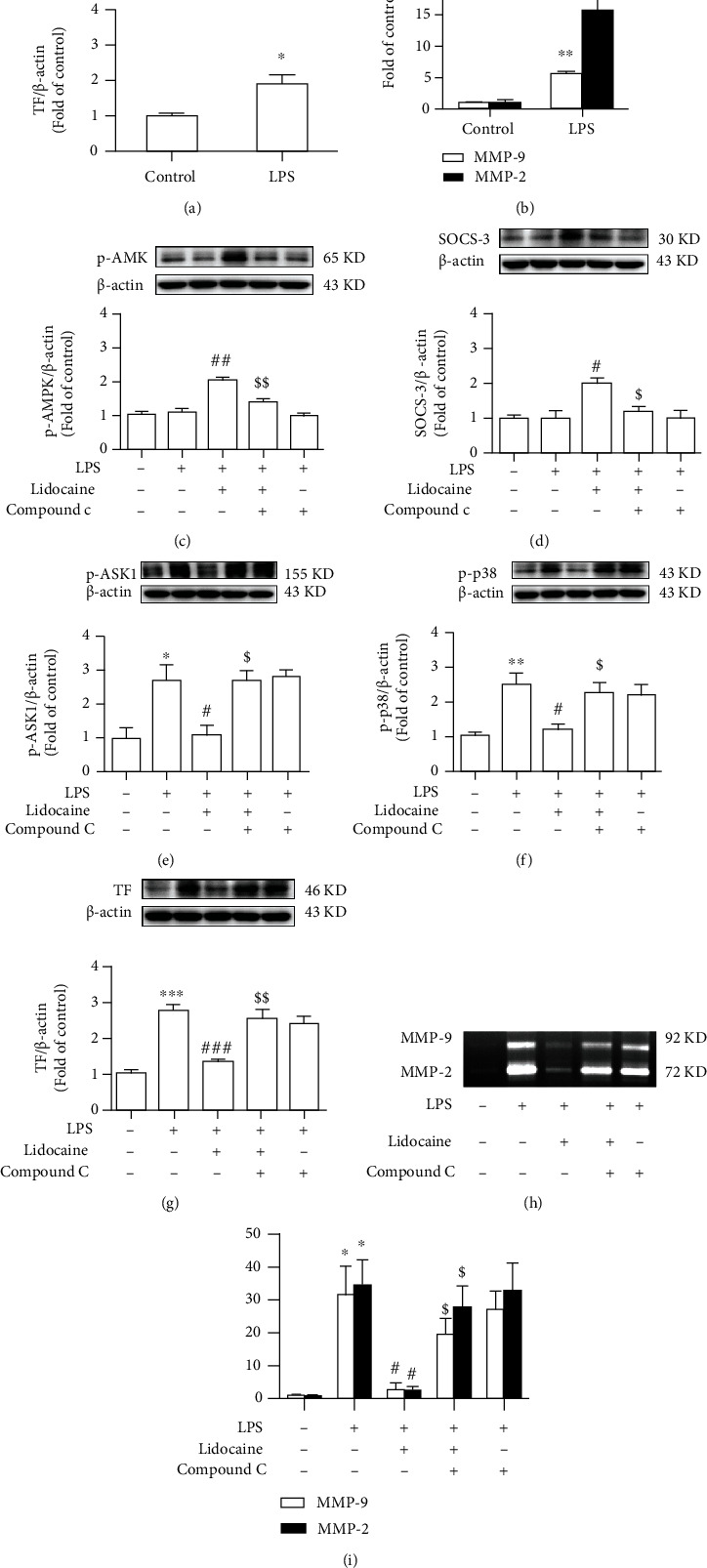
Lidocaine suppressed LPS-induced inflammation in an AMPK-SOCS3-dependent manner. (a, b) Representative images showed the expression of TF in cells and the activity of MMP-2/9 in supernatants. THP1 cells were stimulated with LPS (100 ng/mL) for 12 h (*n* = 3 of each group). (c–g) Representative Western blot bands showed the expression of p-AMPK, SOCS3, p-ASK1, p-p38, and TF in THP1 cells. (h, i) Activity of MMP-2/9 in the supernatants was measured by gelatin zymography, and the densitometry values were normalized. THP1 cells were pretreated with compound C (10 *μ*M) for half an hour before lidocaine treatment, and then, cells were cultured with lidocaine (50 *μ*M) for 6 h, followed by LPS treatment (100 ng/mL) for another 12 h. Then, the cells were collected and analyzed (*n* = 3 of each group). Significant difference was revealed following one-way or two-way ANOVA (^∗^*P* < 0.05, ^∗∗^*P* < 0.01, and ^∗∗∗^*P* < 0.001 vs. control; ^#^*P* < 0.05, ^##^*P* < 0.01, and ^###^*P* < 0.001 vs. LPS-treated group; ^$^*P* < 0.05 and ^$$^*P* < 0.01 vs. lidocaine and LPS-treated group; Bonferroni post hoc tests).

We further conducted experiments on THP1 cells to investigate whether upregulation of AMPK/SOCS3 could inhibit the acute inflammation response induced by LPS. In THP1 cells *in vitro*, as shown in Figures 6(a) and 6(b), compared with the control group, the expression level of TF induced by LPS (100 ng/mL) was increased approximately 2-fold, and the activities of MMP-2/9 were increased nearly 15.7-fold and 5.6-fold, respectively. Moreover, THP1 cell pretreatment with lidocaine (50 *μ*M) for 6 h could increase the expression level of p-AMPK and SOCS3 *in vitro* (Figures 6(c) and 6(d)), whereas preadministration of AMPK inhibitor (compound C, 10 *μ*M) 30 min before LPS stimulation could significantly reduce the expression level of p-AMPK and SOCS3 from 2.01 ± 0.08-fold to 1.35 ± 0.11-fold and from 2.01 ± 0.14-fold to 1.20 ± 0.18-fold, respectively (Figures 6(c) and 6(d)). Besides, lidocaine also effectively decreased the expression of p-ASK1, p-p38, and TF and the activity of MMP-2/9, while pretreatment with compound C (10 *μ*M) could abolish the inhibition of lidocaine on these proteins (Figures 6(e)–6(i)).

## 4. Discussion

In this study, we found that lidocaine could increase the survival rate and weight of LPS-treated mice and reduce the pulmonary edema. We also found that lidocaine could significantly reduce the formation of pulmonary thrombosis in LPS-treated mice, increase blood flow velocity in the subclavian artery, and ameliorate the histopathological damage. Further research show that lidocaine could significantly inhibit the LPS-induced activation of the ASK1-p38-TF/MMP-2/9 signaling pathway *in vivo* and *in vitro*, which depends on AMPK-SOCS3 axis. These results indicated that lidocaine may alleviate sepsis-induced ALI by activating AMPK-SOCS3 axis to inhibit the ASK1-p38-TF/MMP-2/9 signaling pathway.

Acute lung injury (ALI) was first defined in 1967 [23] and characterized as excessive pulmonary inflammation, alveolar-capillary barrier disruption, and pulmonary edema [24]. Numerous studies have demonstrated that in the early stages of infection-induced sepsis ALI, circulating neutrophils infiltrate the lung interstitial and the alveolar cavity to clear microbial pathogens; however, sustained large neutrophil infiltrations could also exacerbate lung damage [25, 26]. There have also been reported that neutrophils have been detected in BALF of acute respiratory distress syndrome (ARDS)/ALI patients, and the number of neutrophils is positively correlated with the severity of lung injury prognosis [27, 28], and the conditional knockout of neutrophils in LPS-treated mice could significantly reduce pulmonary edema and improve ALI [29]. In this study, we also found that the total protein content in BALF was significantly increased in LPS-treated mice (Figure 1(e)), and a large number of neutrophils were detected in the blood (Figures 1(f) and 1(g)). Besides, pathological tissue section results also found that a large number of neutrophils infiltrated into the lung interstitial; interestingly, these effects could be reversed by lidocaine (Figure 2(c)). Study showed that neutrophils could release a large number of inflammatory cytokines and MMPs [30]. MMPs are one of the endopeptidases that could specifically degrade extracellular matrix and chemical or physical barriers, such as blood-brain barrier and vascular nerve barrier. Our previous study found that paraquat could significantly increase the activity of MMP-9 in the lung tissue of mice [29], and evidences have also shown that MMP-9 could in turn exacerbate lung damage [31]. In this study, we also found that the activity of MMP-2/9 was increased in the plasma, BALF, and lung tissue of LPS-treated mice (Figures 3(b), 3(c), and 3(d) and 4(e) and 4(f)), which may be the cause of alveolar capillary barrier disruption and pulmonary edema. Lidocaine that was administrated half an hour earlier could significantly reduce the activity of MMP-2/9 and alleviate pulmonary edema. In addition, it was also found that LPS could significantly increase the activity of MMP-2/9 in THP1 cells *in vitro* (Figure 6(b)), and these effects could be reversed by lidocaine (Figures 6(h) and 6(i)).

Previous study has indicated that coagulopathy appeared in the circulation of patients with sepsis [32, 33], and clinical autopsy reports have also reported fibrin microthrombus in the alveoli and pulmonary vessels of patients with ARDS [34]. Inflammatory stimuli can induce a hypercoagulable state by inducing TF in monocytes and endothelial cells [35]. TF is responsible for triggering the clotting cascade in a variety of thrombotic disorders, thus using anticoagulants, such as tissue factor pathway inhibitor (TFPI) or antithrombin that may be useful in the treatment of ALI and ARDS. TFPI, as an endogenous inhibitor of TF, could directly inhibit activated factor X and, in a factor-dependent manner, produce feedback inhibition of the factor VIIa/TF complex. Evidences also indicated that the lower levels of TFPI are strongly correlated with organ dysfunction as well as worse outcome of severe sepsis [36]. However, activated MMP-9 can degrade TFPI, resulting in the imbalance between TF and TFPI [37]. In addition, the formation of thrombus in the microvascular circulation could also induce the increase of HIF-1*α* expression [38–40], and studies have found that HIF-1*α* can also mediate the expression of MMP-9 [41]. These would further aggravate sepsis-induced ALI by forming a vicious cycle. In this study, our results showed that LPS could induce the formation of pulmonary thrombosis in LPS-treated mice and reduce blood flow velocity in the subclavian artery, whereas this effect could be abolished by lidocaine (Figures 2(a)–2(c)). Besides, LPS could also significantly increase the expression of TF in the plasma and lung tissues of mice in the model group (Figures 3(a) and 4(c)), and this was also confirmed in THP1 cells *in vitro* (Figures 6(a) and 6(g)). However, preadministration of lidocaine significantly reduced the LPS-induced increasing of TF in plasma and lung tissue (Figures 3(a) and 4(c)) and inhibited the expression of HIF-1*α* in lung tissue (Figure 4(d)). Altogether, these data show that lidocaine could significantly inhibit the expression of TF and MMP-2/9 induced by LPS and reduce the formation of pulmonary thrombosis.

**Figure 7:**
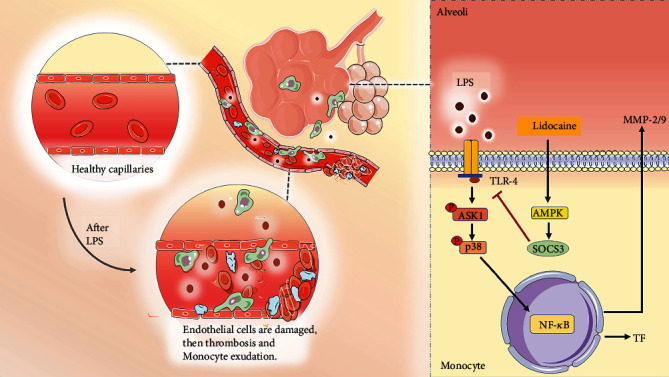
Lidocaine-induced inflammation tolerance alleviates LPS-induced acute lung injury. LPS leads to upregulation of tissue factor (TF) and MMP-2/9 via the TLR4-ASK1-p38 signaling pathway and results in endothelial injury, a hypercoagulable state in the blood vessels, and robust inflammatory infiltration in the lung. Lidocaine pretreatment activates AMPK/SOCS3 axis, which induces inflammatory tolerance and alleviates acute lung injury in mice.

We further investigated the way how lidocaine exerted anti-inflammatory and anticoagulant effects to reduce ALI caused by sepsis. Previous studies have found that ASK1 is essential for the formation of thrombus, and conditional knockout of ASK1 or the use of pharmacologic inhibitors of ASK1 can significantly prolong the bleeding time of mice [10]. In addition, LPS can also induce the increase of TF expression by mediating the activation of the ASK1-p38 signaling pathway [3]. In this study, we also found that LPS significantly induce the expression of p-ASK1, p-p38, and TF in lung tissue, and preadministration of lidocaine at different concentrations (4 and 8 mg/kg) could inhibit these effects (Figures 4(a)–4(c)). As an important negative regulator of cytokine signaling pathways, SOCS3 plays a vital role in a variety of inflammatory diseases, such as multiple sclerosis [42], experimental autoimmune encephalomyelitis [43], pain [15], and airway inflammation [44]. Moreover, several studies have found that overexpression of SOCS3 could simultaneously inhibit a variety of inflammatory signals, including TLR4, TNF-R, IL-1R, and IL-6R [45, 46]. Additionally, our previous research also indicated that lidocaine could induce the upregulation of SOCS3, and using SOCS3 small interfering RNA to knockdown SOCS3 sufficiently abolished anti-inflammatory effects of lidocaine; besides, we also found that lidocaine induced upregulation of SOCS3 expression in an AMPK-dependent manner [17], and recent studies have also attempted to use the anti-inflammatory cytokines, such as IL-4, IL-10, and IL-13, to inhibit TF expression in monocytes and protect against ALI [47]. In this study, we further investigated whether lidocaine can mediate AMPK activation and SOCS3 expression in LPS-induced acute lung injury model and whether lidocaine can alleviate LPS-mediated pulmonary edema, coagulation dysfunction, and inflammatory response. Our results show that lidocaine could significantly reduce the level of IL-1*β*, TNF-*α*, and IL-6 in the serum and lung tissue of LPS-treated mice (Figure S2). Moreover, lidocaine could also significantly induce the expression of p-AMPK and SOCS3 *in vitro* and *in vivo* (Figure 5, Figures 6(c) and 6(d), and Figure S3a-c). Meanwhile, preadministration of AMPK inhibitor (compound C) could reduce the expression of p-AMPK and SOCS3 (Figures 6(c) and 6(d) and Figure S3a-c) and abolish the inhibition of lidocaine on ASK1, p38, and TF *in vitro* (Figures 6(e)–6(g) and Figure S3d-f). Taken together, these results indicate that lidocaine may promote the expression of SOCS3 in an AMPK-dependent manner, inhibiting the activation of the ASK1-p38-TF/MMP-2/9 signaling pathway to decrease the formation of thrombus and to alleviate sepsis-induced ALI (Figure 7).

In conclusion, we provided the experimental evidence that lidocaine could alleviate sepsis-induced ALI via activating AMPK/SOCS3 axis to inhibit the ASK1-p38-TF/MMP-2/9 signaling pathway. Our findings further enriched the pathogenesis of ALI and provide a potential treatment for ALI.

## Data Availability

The data used to support the findings of this study are available from the corresponding authors with reasonable request.

## Supplementary Materials

Supplementary Materials The supplementary material for this article can be found online. Figure S1: the plasma drug concentration of lidocaine at various time points. Figure S2: lidocaine significantly suppressed LPS-induced inflammatory response in mice. Figure S3: lidocaine alleviated sepsis-induced acute lung injury via activating AMPK/SOCS3 axis to inhibit the ASK1-p38-TF/MMP-2/9 signaling pathway in MLE-12 cells *in vitro*. Table S1: pharmacokinetic variables of lidocaine following i.v. administration of 8 mg/kg of lidocaine for 12 h to mice (mean ± SD, *n* = 3).

## References

[1] Abraham E. (2000). Coagulation abnormalities in acute lung injury and sepsis. *American Journal of Respiratory Cell and Molecular Biology*.

[2] Chen X., Lao Y., Yi J., Yang J., He S., Chen Y. (2020). SENP3 in monocytes/macrophages up-regulates tissue factor and mediates lipopolysaccharide-induced acute lung injury by enhancing JNK phosphorylation. *Journal of Cellular and Molecular Medicine*.

[3] Mizumura K., Gon Y., Kumasawa F. (2010). Apoptosis signal-regulating kinase 1-mediated signaling pathway regulates lipopolysaccharide-induced tissue factor expression in pulmonary microvasculature. *International Immunopharmacology*.

[4] Carraway M. S., Welty-Wolf K. E., Miller D. L. (2003). Blockade of tissue factor: treatment for organ injury in established sepsis. *American Journal of Respiratory and Critical Care Medicine*.

[5] Yi L., Huang X., Guo F., Zhou Z., Dou Y., Huan J. (2016). Yes-associated protein (YAP) signaling regulates lipopolysaccharide-induced tissue factor expression in human endothelial cells. *Surgery*.

[6] Chen D., Giannopoulos K., Shiels P. G. (2004). Inhibition of intravascular thrombosis in murine endotoxemia by targeted expression of hirudin and tissue factor pathway inhibitor analogs to activated endothelium. *Blood*.

[7] Matsuzawa A., Nishitoh H., Tobiume K., Takeda K., Ichijo H. (2002). Physiological roles of ASK1-mediated signal transduction in oxidative stress- and endoplasmic reticulum stress-induced apoptosis: advanced findings from ASK1 knockout mice. *Antioxidants & Redox Signaling*.

[8] Takeda K., Matsuzawa A., Nishitoh H., Ichijo H. (2003). Roles of MAPKKK ASK1 in stress-induced cell death. *Cell Structure and Function*.

[9] Ansari S. A., Pendurthi U. R., Rao L. V. M. (2017). The lipid peroxidation product 4-hydroxy-2-nonenal induces tissue factor decryption via ROS generation and the thioredoxin system. *Blood Advances*.

[10] Naik M. U., Patel P., Derstine R. (2017). Ask1 regulates murine platelet granule secretion, thromboxane A2 generation, and thrombus formation. *Blood*.

[11] Tong W., Chen X., Song X. (2020). Resveratrol inhibits LPS-induced inflammation through suppressing the signaling cascades of TLR4-NF-*κ*B/MAPKs/IRF3. *Experimental and Therapeutic Medicine*.

[12] Gao H., Kang N., Hu C. (2020). Ginsenoside Rb1 exerts anti-inflammatory effects *in vitro* and *in vivo* by modulating Toll-like receptor 4 dimerization and NF-kB/MAPKs signaling pathways. *Phytomedicine*.

[13] Park J., Ha S. H., Abekura F. (2019). 4-O-Carboxymethylascochlorin inhibits expression levels of on inflammation-related cytokines and matrix metalloproteinase-9 through NF-kappaB/MAPK/TLR4 signaling pathway in LPS-activated RAW264.7 cells. *Frontiers in Pharmacology*.

[14] Kubo M., Hanada T., Yoshimura A. (2003). Suppressors of cytokine signaling and immunity. *Nature Immunology*.

[15] Fan Y. X., Qian C., Liu B. (2018). Induction of suppressor of cytokine signaling 3 via HSF-1-HSP70-TLR4 axis attenuates neuroinflammation and ameliorates postoperative pain. *Brain, Behavior, and Immunity*.

[16] Kim J. E., Kim K. J., Ahn W., Han K. S., Kim H. K. (2011). Local anesthetics inhibit tissue factor expression in activated monocytes via inhibition of tissue factor mRNA synthesis. *Clinical and Applied Thrombosis/Hemostasis*.

[17] Zhang Y., Tao G. J., Hu L. (2017). Lidocaine alleviates morphine tolerance via AMPK-SOCS3-dependent neuroinflammation suppression in the spinal cord. *Journal of Neuroinflammation*.

[18] Cao F., Tian X., Li Z. (2020). Suppression of NLRP3 inflammasome by erythropoietin via the EPOR/JAK2/STAT3 pathway contributes to attenuation of acute lung injury in mice. *Frontiers in Pharmacology*.

[19] Kim K. H., Kwun M. J., Choi J. Y. (2013). Therapeutic Effect of the Tuber of Alisma orientale on Lipopolysaccharide- Induced Acute Lung Injury. *Evidence-Based Complementary and Alternative Medicine*.

[20] Jeong S. Y., Kim J., Park E. K., Baek M. C., Bae J. S. (2020). Inhibitory functions of maslinic acid on particulate matter-induced lung injury through TLR4-mTOR-autophagy pathways. *Environmental Research*.

[21] Levi M., van der Poll T. (2017). Coagulation and sepsis. *Thrombosis Research*.

[22] Shaver C. M., Grove B. S., Putz N. D. (2015). Regulation of alveolar procoagulant activity and permeability in direct acute lung injury by lung epithelial tissue factor. *American Journal of Respiratory Cell and Molecular Biology*.

[23] Ashbaugh D. G., Bigelow D. B., Petty T. L., Levine B. E. (1967). Acute respiratory distress in adults. *Lancet*.

[24] Matthay M. A., Zemans R. L., Zimmerman G. A. (2019). Acute respiratory distress syndrome. *Nature Reviews. Disease Primers*.

[25] Sekheri M., El Kebir D., Edner N., Filep J. G. (2020). 15-Epi-LXA4 and 17-epi-RvD1 restore TLR9-mediated impaired neutrophil phagocytosis and accelerate resolution of lung inflammation. *Proceedings of the National Academy of Sciences of the United States of America*.

[26] Giacalone V. D., Margaroli C., Mall M. A., Tirouvanziam R. (2020). Neutrophil adaptations upon recruitment to the lung: new concepts and implications for homeostasis and disease. *International Journal of Molecular Sciences*.

[27] Stapleton R. D., Suratt B. T., Neff M. J. (2019). Bronchoalveolar fluid and plasma inflammatory biomarkers in contemporary ARDS patients. *Biomarkers*.

[28] Grommes J., Soehnlein O. (2011). Contribution of neutrophils to acute lung injury. *Molecular Medicine*.

[29] Zhang F., Hu L., Wu Y. X. (2019). Doxycycline alleviates paraquat-induced acute lung injury by inhibiting neutrophil-derived matrix metalloproteinase 9. *International Immunopharmacology*.

[30] Potey P. M., Rossi A. G., Lucas C. D., Dorward D. A. (2019). Neutrophils in the initiation and resolution of acute pulmonary inflammation: understanding biological function and therapeutic potential. *The Journal of Pathology*.

[31] Jan J. S., Yang C. H., Wang M. H. (2019). Hirsutanol A attenuates lipopolysaccharide-mediated matrix metalloproteinase 9 expression and cytokines production and improves endotoxemia-induced acute sickness behavior and acute lung injury. *Marine Drugs*.

[32] Simmons J., Pittet J. F. (2015). The coagulopathy of acute sepsis. *Current Opinion in Anaesthesiology*.

[33] Vincent J. L., Francois B., Zabolotskikh I. (2019). Effect of a recombinant human soluble thrombomodulin on mortality in patients with sepsis-associated coagulopathy: the SCARLET randomized clinical trial. *JAMA*.

[34] Bone R. C., Francis P. B., Pierce A. K. (1976). Intravascular coagulation associated with the adult respiratory distress syndrome. *The American Journal of Medicine*.

[35] Xue M., Sun Z., Shao M. (2015). Diagnostic and prognostic utility of tissue factor for severe sepsis and sepsis-induced acute lung injury. *Journal of Translational Medicine*.

[36] Tang H., Ivanciu L., Popescu N. (2007). Sepsis-induced coagulation in the baboon lung is associated with decreased tissue factor pathway inhibitor. *The American Journal of Pathology*.

[37] Belaaouaj A. A., Li A., Wun T. C., Welgus H. G., Shapiro S. D. (2000). Matrix Metalloproteinases Cleave Tissue Factor Pathway Inhibitor:. *Journal of biological chemistry*.

[38] Morishige K., Shimokawa H., Matsumoto Y. (2003). Overexpression of matrix metalloproteinase-9 promotes intravascular thrombus formation in porcine coronary arteries in vivo. *Cardiovascular Research*.

[39] Semenza G. L. (2010). Vascular responses to hypoxia and ischemia. *Arteriosclerosis, Thrombosis, and Vascular Biology*.

[40] Kasivisvanathan V., Shalhoub J., Lim C., Shepherd A., Thapar A., Davies A. (2011). Hypoxia-inducible factor-1 in arterial disease: a putative therapeutic target. *Current Vascular Pharmacology*.

[41] Choi J. Y., Jang Y. S., Min S. Y., Song J. Y. (2011). Overexpression of MMP-9 and HIF-1*α* in breast cancer cells under hypoxic conditions. *Journal of Breast Cancer*.

[42] Qin H., Yeh W. I., de Sarno P. (2012). Signal transducer and activator of transcription-3/suppressor of cytokine signaling-3 (STAT3/SOCS3) axis in myeloid cells regulates neuroinflammation. *Proceedings of the National Academy of Sciences of the United States of America*.

[43] Liu Y., Holdbrooks A. T., Meares G. P., Buckley J. A., Benveniste E. N., Qin H. (2015). Preferential recruitment of neutrophils into the cerebellum and brainstem contributes to the atypical experimental autoimmune encephalomyelitis phenotype. *Journal of Immunology*.

[44] Draijer C., Speth J. M., Penke L. R. K. (2020). Resident alveolar macrophage-derived vesicular SOCS3 dampens allergic airway inflammation. *The FASEB Journal*.

[45] Croker B. A., Krebs D. L., Zhang J. G. (2003). SOCS3 negatively regulates IL-6 signaling in vivo. *Nature Immunology*.

[46] Frobøse H., Groth Rønn S., Heding P. E. (2006). Suppressor of cytokine signaling-3 inhibits interleukin-1 signaling by targeting the TRAF-6/TAK1 complex. *Molecular Endocrinology*.

[47] Osnes L. T., Westvik A. B., Joø G. B., Okkenhaug C., Kierulf P. (1996). Inhibition of IL-1 induced tissue factor (TF) synthesis and procoagulant activity (PCA) in purified human monocytes by IL-4, IL-10 and IL-13. *Cytokine*.

