# Supplementary material for: Lidocaine Alleviates Sepsis-Induced Acute Lung Injury in Mice by Suppressing Tissue Factor and Matrix Metalloproteinase-2/9
Source: Oxid Med Cell Longev. 2021 Nov 12;2021:3827501. doi: 10.1155/2021/3827501 (PMC8604580; doi:10.1155/2021/3827501)
Supplement: Supplementary Materials — The supplementary material for this article can be found online. Figure S1: the plasma drug concentration of lidocaine at various time points. Figure S2: lidocaine significantly suppressed LPS-induced inflammatory response in mice. Figure S3: lidocaine alleviated sepsis-induced acute lung injury via activating AMPK/SOCS3 axis to inhibit the ASK1-p38-TF/MMP-2/9 signaling pathway in MLE-12 cells in vitro. Table S1: pharmacokinetic variables of lidocaine following i.v. administration of 8 mg/kg of lidocaine for 12 h to mice (mean ± SD, n = 3). [file 3827501.f1.docx]

**Lidocaine** **alleviates sepsis-induced** **acute lung injury in mice by suppressing tissue factor and matrix metalloproteinase-2/9**

Binbin Zheng^1, 2, 3, ϯ^, Hongbo Yang^1, 2, 3, ϯ^, Jianan Zhang^4, ϯ^, Xueli Wang^1, 2, 3, ϯ^, Hao Sun^5^, Fan Hu^4^, Qian Li^6^, Liping Jiang^6^, Yue Su^4^, Qilin Peng^4^, Yulin Tang^7^, Wen-Tao Liu^3, 4^, Xueming He^1, 2, 3, 🖂^, Yixin Fan^4, 7, 🖂^, Xia Zhu^1, 2, 3, 🖂^

^1^ Center for Clinical Research and Translational Medicine, The Affiliated Lianyungang Oriental Hospital of Xuzhou Medical University, Lianyungang 222042, China

^2^ Center for Clinical Research and Translational Medicine, The Affiliated Lianyungang Oriental Hospital of Bengbu Medical College, Lianyungang 222042, China

^3^ Center for Clinical Research and Translational Medicine, The Affiliated Lianyungang Oriental Hospital of Kangda College of Nanjing Medical University, Lianyungang 222042, China

^4^ Jiangsu Key Laboratory of Neurodegeneration, Department of Pharmacology, Nanjing Medical University, Nanjing 211166, China

^5^ Department of Emergency, Jiangsu Province Hospital, The First Affiliated Hospital of Nanjing Medical University, Nanjing 210029, China

^6^ Department of Anesthesiology, The affiliated Jiangning Hospital of Nanjing Medical University, Nanjing, Jiangsu, China

^7^ Department of Pharmacy, Sir Run Run Hospital, Nanjing Medical University, No.109 Longmian Avenue, Nanjing 211100, China

**^ϯ^** These authors contributed equally to this work.

^🖂^ Correspondence author.

Correspondence author at: Center for Clinical Research and Translational Medicine, The Affiliated Lianyungang Oriental Hospital of Xuzhou Medical University, Lianyungang 222042, China

Or

Department of Pharmacy, Sir Run Run Hospital, Nanjing Medical University, No.109 Longmian Avenue, Nanjing 211100, China

Or

Center for Clinical Research and Translational Medicine, The Affiliated Lianyungang Oriental Hospital of Xuzhou Medical University, Lianyungang 222042, China

*E-mail address*: 15261379088@139.com (X-M. He); L_fan1991@163.com (Y-X. Fan); zhuxiadfyy@163.com (X. Z)

**Supplementary Data**


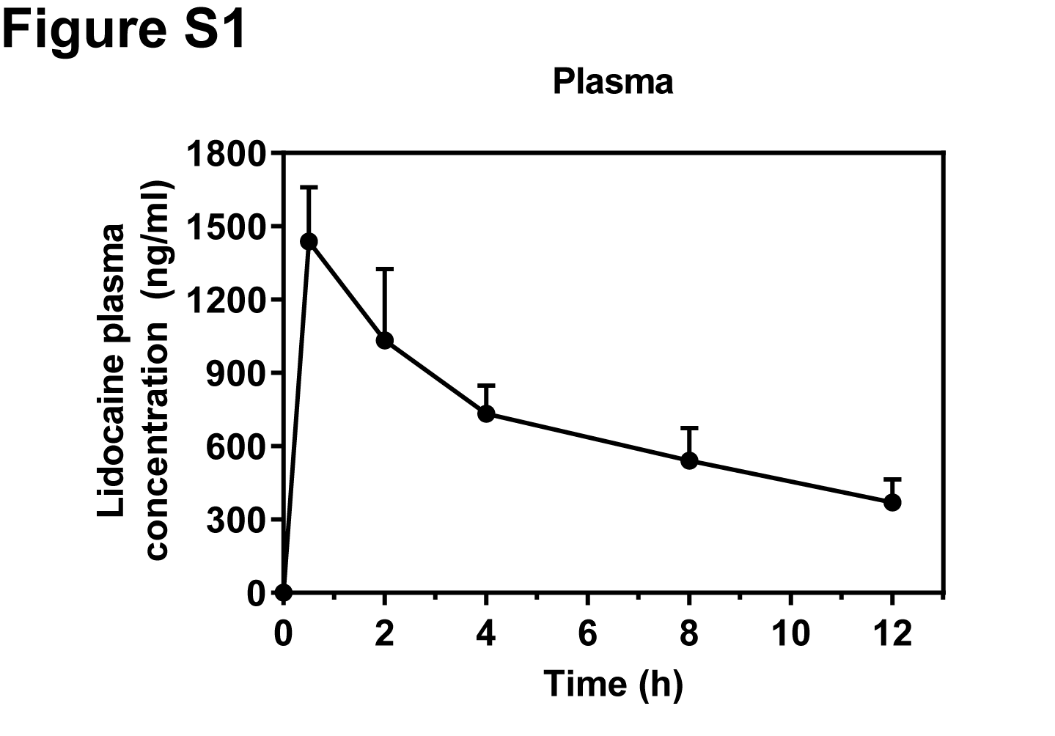


Figure S1. The plasma drug concentration of lidocaine at various time points after *i.v.* administration of a single dose of lidocaine hydrochloride (8 mg/kg) to mice.

Table S1 Pharmacokinetic variables of lidocaine following *i.v.* administration of 8 mg/kg of lidocaine for 12 h to mice. (mean ± SD, n = 3)

| C_max_(ng/ml) | T_max_(h) | AUC (ng×h/ml) | t_1/2_(h) |
| --- | --- | --- | --- |
| 1437. 7 ± 221.4 | 0.5 ± 0.08 | 8344 ± 641.2 | 1.6 ± 0.2 |

C_max_ = Maximum observed plasma drug concentration; T_max_ = Time after lidocaine administration at which C_max_ was observed; AUC = Area under the plasma drug concentration-versus-time curve; t_1/2_(h) = Elimination half-life.


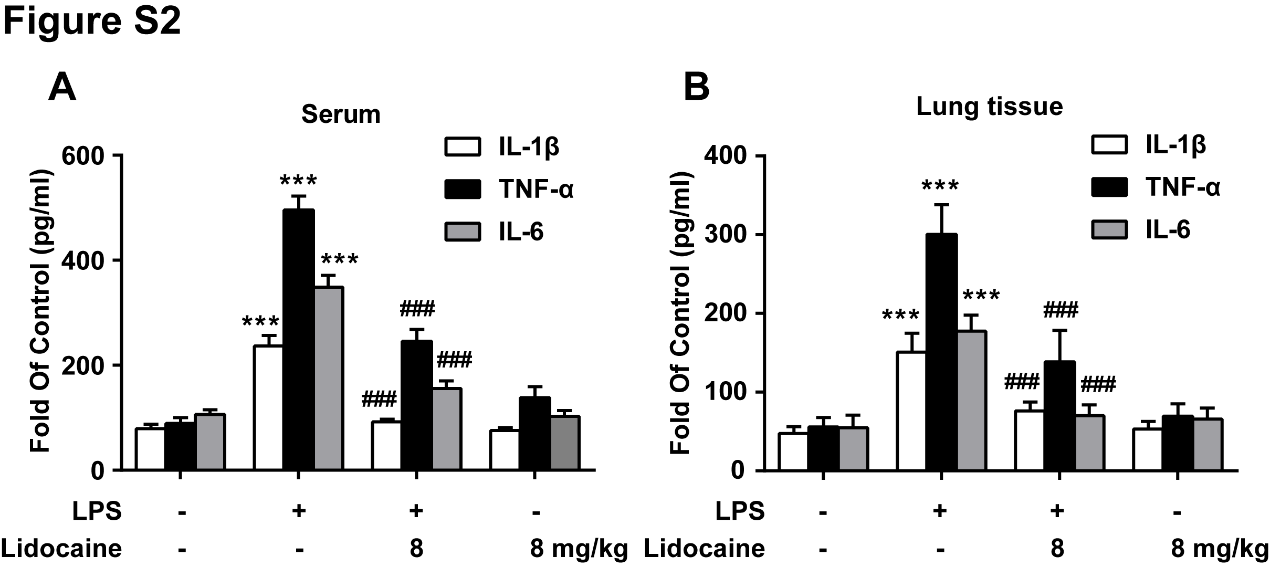


Figure S2. Lidocaine significantly suppressed LPS-induced inflammatory response in mice. (A) Effect of lidocaine on the expression of IL-1β, TNF-α and IL-6 in serum was measured by Elisa kits. (B) Effect of lidocaine on the expression of IL-1β, TNF-α and IL-6 in the lung tissue was measured by Elisa kits. Mice were treated with lidocaine (8 mg/kg, *i.v.*) 30 min before LPS injection, and the serum and lung tissue samples (n = 3 of each group) were collected 12 h after LPS given. Significant difference was revealed following one-way ANOVA (^***^*P* < 0.001 vs. control; ^###^*P* < 0.001 vs. LPS-treated group; Bonferroni post hoc tests).


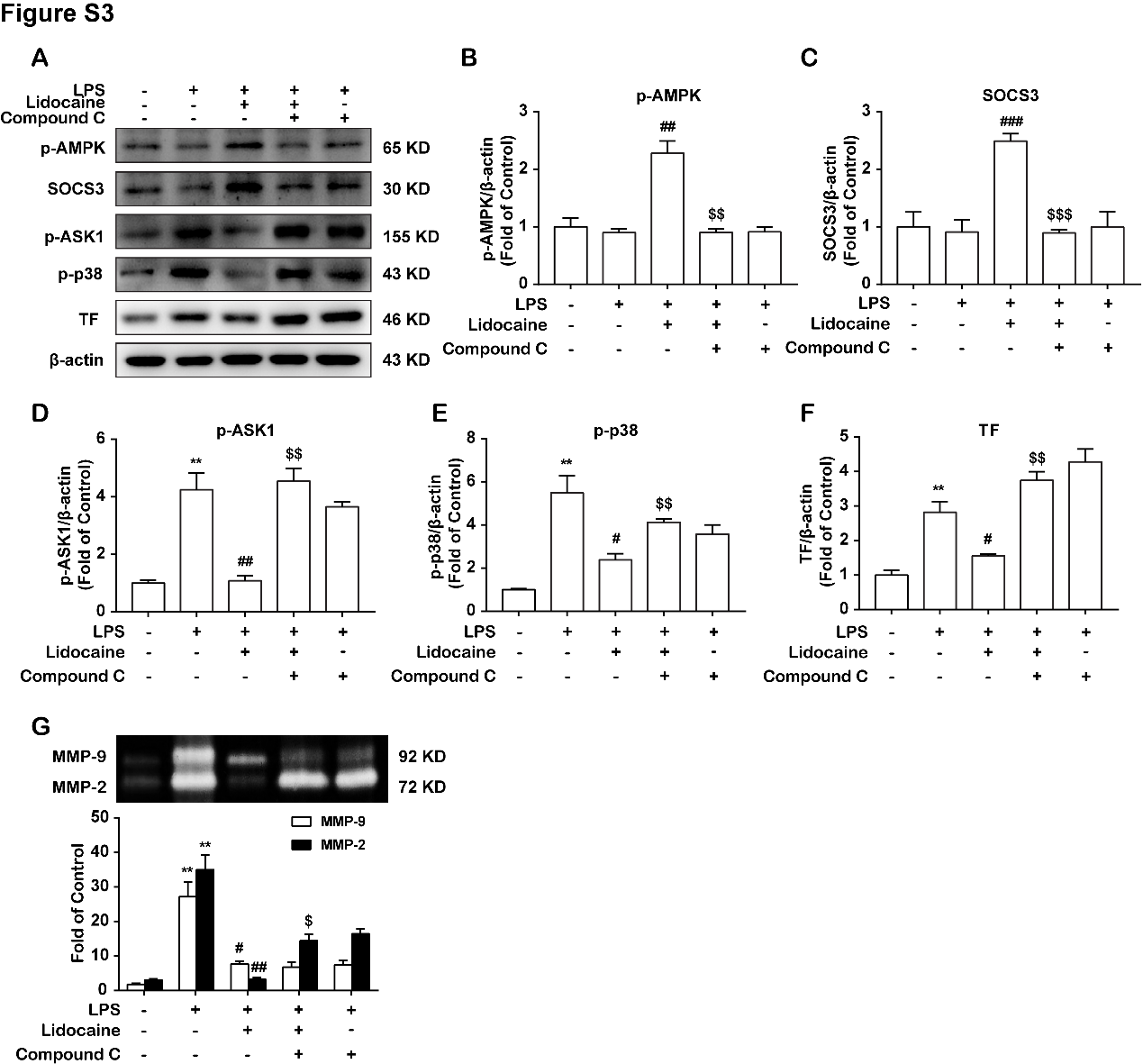


Figure S3. Lidocaine alleviated sepsis-induced acute lung injury via activating AMPK/SOCS3 axis to inhibit ASK1-p38-TF/MMP-2/9 signaling pathway in MLE-12 cells *in vitro*. (A-F) Representative Western blot bands showed the expression of p-AMPK, SOCS3, p-ASK1 p-p38 and TF in MLE-12 cells *in vitro*. (G) Activity of MMP-2/9 in the supernatants was measured by gelatin zymography and the densitometry values were normalized. MLE-12 cells were pretreated with Compound C (10 μM) for half an hour before lidocaine treatment, and then cells were cultured with lidocaine (50 μM) for 6 h, followed by LPS treatment (100 ng/mL) for another 12 h. Then, the cells were collected and analyzed (n = 3 of each group). Significant difference was revealed following one-way or two-way ANOVA (^**^*P* < 0.01 vs. control; ^#^*P* < 0.05, ^##^*P* < 0.01, ^###^*P* < 0.001 vs. LPS-treated group; ^$^*P* < 0.05, ^$$^*P* < 0.01, ^$$$^*P* < 0.001 vs. lidocaine and LPS-treated group; Bonferroni post hoc tests).
